# Experimental data of heat transfer nanofluids for trigeneration systems: viscosity at below-ambient temperatures

**DOI:** 10.1016/j.dib.2022.108854

**Published:** 2023-01-14

**Authors:** Guilherme Cunha Maia Nobre, Luiz Umberto Rodrigues Sica, Edwin Martin Cardenas Contreras, Enio Pedone Bandarra Filho, Paul Ortega Sotomayor, José Alberto Reis Parise

**Affiliations:** a Department of Mechanical Engineering, Pontifícia Universidade Católica do Rio de Janeiro, Rua Marquês de São Vicente 225, Rio de Janeiro, RJ 22451-900, Brazil; b Centro de Tecnologia, bloco A, Cidade Universitária, Institute of Physics, Universidade Federal do Rio de Janeiro, Av. Athos da Silveira Ramos, 149, Rio de Janeiro, RJ 21941-972, Brazil; c School of Mechanical Engineering, Universidade Federal de Uberlândia, Av. João Naves de Ávila, 2121, Uberlândia, MG 38400-902, Brazil; d The Tecgraf Institute of Technical-Scientific Software Development at PUC-Rio, Bldg. Pe. Laércio Dias de Moura, Rua Marquês de São Vicente 225, Rio de Janeiro, RJ 22451-900, Brazil

**Keywords:** Nanofluids, Graphene, MWCNT- multi walled carbon nano tube, CCHP – combined cooling heating and power, Dynamic viscosity, Below ambient temperatures, ASTM, American Society for Testing and Materials, CAPES, Coordenação de Aperfeiçoamento de Pessoal de Nível Superior, CCHP, Combined Cooling Heating and Power, CNPq, Conselho Nacional de Desenvolvimento Científico e Tecnológico, CNT, Carbon Nano Tube, FAPEMIG, Fundação de Amparo à Pesquisa do Estado de Minas Gerais, LRAC, Laboratório de Refrigeração e Condcionamento de Ar, FAPERJ, Fundação de Amparo à Pesquisa do Estado do Rio de Janeiro, LEST-nano, Energy, Thermal Systems and Nanotechnology Laboratory at UFU, MCNT, MultiWalled Carbon Nano Tube, NBR, Normas Brsileiras, PUC-Rio, Pontifícia Universidade Católica do Rio de Janeiro, UFU, Federal University of Uberlândia

## Abstract

The present work exhibits the dynamic viscosity profile data of three distinct nanofluids, at a constant shear stress, and within a range of temperatures that include below-ambient conditions (from −10 to 20 °C). The nanofluids were as follows. Nanofluid I: 30% ethylene glycol and 70% distilled water (v/v), with graphene (0.32% in mass); Nanofluid II: 30% engine coolant NBR 13705; ASTM D-3306; ASTM D-4985) and 70% distilled water (v/v), with graphene (0.2% in mass); and Nanofluid III: 30% engine coolant and 70% distilled water (v/v), with Multi-Walled Carbon Nanotubes (MWCNT) (0.2% in mass). The present work was motivated by the scarcity of experimental data on the temperature dependence of viscosity for graphene, MWCNT, and their hybrid nanofluids, at below-ambient temperatures.

**Specifications Table**

**Table:** 

Subject	Mechanical Engineering
Specific subject area	Thermal Engineering
Type of data	Table
How the data were acquired	The data were acquired by means of a viscometer (V2-L/ VR 3000 MYR) and a thermostatic bath (Visomes Plus, VBT 2050). Given the relatively small number of variables and experimental runs, data acquisition was carried out manually, with a numerical spreadsheets being fed by instrument readings. Further details can be found in section “Experimental Design, Materials and Methods”, below.
Data format	Raw data
Description of data collection	Viscosity measurements of three specific nanofluids were carried out in a temperature-controlled environment by means of a rotational viscometer and a thermostatic bath. The nanofluid sample temperature was stabilized for 30 minutes, by adjusting the thermal bath temperature at a prescribed value. Shear stress was also stabilized resulting in the shear rate as a fixed experimental parameter.
Data accessibility	(1) Name of the repository: Mendeley (2) Data Identification Number: 10.17632/5xz8jrcmkb.1 (3) Direct URL to the data: https://data.mendeley.com/datasets/5xz8jrcmkb/1
Related research article	[1] L.U.R. Sica, G.C.M. Nobre, E.M. Contreras, E.P Bandarra Filho, P.O. Sotomayor, J.A.R. Parise, Heat transfer nanofluids for trigeneration systems: fabrication and experimental investigation of viscosity at below -ambient temperatures, International Journal of Refrigeration 129 (2021) 163-174. 10.1016/j.ijrefrig.2021.04.036

## Value of the Data

• The usefulness of the data relies on the fact that nanofluids usually present enhanced viscosity [2] causing a negative effect on the performance of trigeneration plants. This effect would be governed by several design parameters (temperature range; type, material, geometry, and concentration of nanoparticles; base fluid properties), a few of which have been varied in the experiment.
• Researchers and thermal engineers involved with the modelling and design of trigeneration plants would most benefit from the data here presented. Viscosity is a key parameter in the calculation of pressure drop and heat transfer parameters.
• The literature, for example [3,4], has shown that the methodology adopted for the calculation of pressure drop and heat transfer of Newtonian fluids can be extended to Newtonian nanofluids, provided their thermophysical properties and specific correlations are used. Therefore, the data here presented allow for a valuable and original information on the viscosity of graphene and MWCNT nanofluids, particularly at below-ambient temperatures, inherent to refrigeration, and trigeneration systems, as well.
• Due to their observed enhancement in thermophysical properties, heat transfer nanofluids, engineered colloidal suspensions of solid nanoparticles in a heat transfer base fluid, for example, [5,6], can potentially improve the performance of several thermal systems, including trigeneration plants (below-ambient temperature fluid flowing between refrigeration plant and cold demand point).
• Given the scarcity of thermophysical properties related to these nanofluids, notably at below-ambient temperatures, the data presented here will be useful and important to thermal engineers, as more accurate results for the calculation of thermal systems, will be available. In this respect, the Reynolds number, which is a dimensionless flow parameter, is strongly influenced by fluid viscosity and is also the control flow parameter in thermal systems that involve heat transfer, fluid flow, and pressure drop. Therefore, refrigeration as well as trigeneration systems may benefit from the use of the experimental data from [1], since a more precise operational condition can be attained. The data can, of course, be re-used for further insight, research, and development, by means of: (i) data points direct collection in Tables 1 to 4, or (ii) by curve fitting equations.

**Table 1:** Raw data of viscometer validation with run number in column 1, sample temperature in column 2, measured viscosity of Mixture I, distilled water and ethylene glycol, (vol 50%-50%), in column 3, and values of viscosity for the same mixture composition, in columns 4, 5 and 6.

Run	Temperature (°C)	Present data -Measured viscosity of Mixture I (mPas)	Melinder [7] viscosity(mPas)	Mirmomohammadi [8] viscosity(mPas)	Huntsman [9] viscosity(mPas)
1	-5	11.2	11.8	-	12.0
2	0	9.5	9.0	-	9.0
3	5	7.3	7.5	-	7.0
4	10	6.2	5.9	-	6.0
5	20	4.7	4.1	4.5	4.4
6	30	3.6	3.2	3.3	3.2
7	40	2.6	2.3	2.5	2.4
8	50	2.0	1.9	2.0	1.8

## 1 Data Description

The present data-article intents to exhibit the temperature dependency of the dynamic viscosity of three distinct nanofluids, each one with constant shear stress, at a range of operation that includes below-ambient temperatures (from −10 to 20 °C). Graphene and MWCNT nanoparticles were dispersed in base-fluids consisting of different mixtures of prime-mover coolant (NBR 13705; ASTM D-3d306; ASTM D-4985), distilled water, and ethylene glycol. First, the verification of the rotational viscometer at below-ambient temperatures was an important step in this research since it enabled the functional verification of the instrument under the conditions of the experimental investigation. It consisted of adjusting the viscometer and then checking its accuracy against reference fluids commonly used in the literature [7], [8], [9]. Raw data are presented in Table 1, with run number in column 1, sample temperature in column 2, measured viscosity (mPas) of Mixture I (distilled water and ethylene glycol, vol 50%-50%), in column 3, and literature [7], [8], [9] values of viscosity for the same mixture composition, in columns 4, 5 and 6. Temperature and viscosity measurements were taken at every 5 °C. The temperature dependency of the dynamic viscosity of three distinct nanofluids is presented in Tables 2, 3 and 4, with constant shear stress, and over a range that includes below-ambient temperatures (from −10 to 20°C), constructed over 35 experimental runs (each one). They are arranged in a similar fashion with run number in column 1, sample temperature (°C) in column 2, and measured viscosities (mPas) of base fluid and nanofluid in columns 3 and 4, respectively. Table 2 shows raw data of measured viscosities (mPas) of base fluid I and nanofluid II. Similarly, Tables 3 and 4 contain raw data of base fluid I plus nanofluid III and base fluid II plus nanofluid I, respectively, in columns 3 and 4, respectively.

**Table 2:** Raw data of viscometer measurements with run number in column 1, sample temperature (°C)) in column 2, and measured viscosities (mPas) of base fluid I and nanofluid II, in columns 3 and 4, respectively.

Run	Temperature (°C)	Viscosity of base fluid I (mPas)	Viscosity of nanofluid II (mPas)
1	-6.0	5.1	7.4
2	-5.6	5.1	7.4
3	-5.6	5.2	7.4
4	-5.6	5.2	7.4
5	-5.6	5.1	7.4
6	-4.2	4.9	7.4
7	-4.6	4.9	7.4
8	-4.6	4.9	7.4
9	-4.6	4.9	7.4
10	-4.6	5.0	7.4
11	0.2	4.2	6.3
12	-0.2	4.2	6.3
13	-0.2	4.3	6.3
14	-0.2	4.3	6.3
15	-0.2	4.2	6.3
16	4.7	3.7	5.9
17	4.7	3.7	5.9
18	4.7	3.7	5.9
19	4.9	3.7	5.9
20	4.9	3.6	5.9
21	10.5	3.2	5.3
22	10.0	3.2	5.3
23	10.3	3.4	5.3
24	10.0	3.3	5.3
25	9.9	3.3	5.3
26	15.0	3.0	4.9
27	14.5	3.0	4.9
28	15.0	3.0	4.9
29	15.0	3.0	4.9
30	15.0	3.0	4.9
31	20.0	2.7	4.6
32	20.0	2.7	4.6
33	20.0	2.6	4.6
34	20.0	2.6	4.6
35	20.0	2.6	4.6

**Table 3:** Raw data of viscometer measurements with run number in column 1, sample temperature (°C) in column 2, and measured viscosities (mPas) of base fluid I and nanofluid III in columns 3 and 4, respectively.

Run	Temperature (°C)	Viscosity of base fluid I (mPas)	Viscosity of nanofluid III (mPas)
1	-6.0	5.1	7.5
2	-5.6	5.1	7.5
3	-5.6	5.2	7.5
4	-5.6	5.2	7.5
5	-5.6	5.1	7.5
6	-4.2	4.9	7.5
7	-4.6	4.9	7.5
8	-4.6	4.9	7.5
9	-4.6	4.9	7.5
10	-4.6	5.0	7.5
11	0.2	4.2	6.3
12	-0.2	4.2	6.3
13	-0.2	4.3	6.3
14	-0.2	4.3	6.3
15	-0.2	4.2	6.3
16	4.7	3.7	5.6
17	4.7	3.7	5.6
18	4.7	3.7	5.6
19	4.9	3.7	5.6
20	4.9	3.6	5.6
21	10.5	3.2	5.4
22	10.0	3.2	5.4
23	10.3	3.4	5.4
24	10.0	3.3	5.4
25	9.9	3.3	5.4
26	15.0	3.0	4.9
27	14.5	3.0	4.9
28	15.0	3.0	4.9
29	15.0	3.0	4.9
30	15.0	3.0	4.9
31	20.0	2.7	4.8
32	20.0	2.7	4.8
33	20.0	2.6	4.8
34	20.0	2.6	4.8
35	20.0	2.6	4.8

**Table 4:** Raw data of viscometer measurements with run number in column 1, sample temperature (°C) in column 2, and measured viscosities (mPas) of base fluid II and nanofluid I, in columns 3 and 4, respectively.

Run	Temperature (°C)	Viscosity of base fluid II (mPas)	Viscosity of nanofluid I (mPas)
1	-10	8.6	10.5
2	-10	8.7	10.5
3	-10	8.6	10.5
4	-10	8.6	10.5
5	-10	8.6	10.5
6	-5	6.7	9.5
7	-5	6.7	9.5
8	-5	6.6	9.5
9	-5	6.6	9.5
10	-5	6.6	9.5
11	0	5.3	8.5
12	0	5.4	8.5
13	0	5.4	8.5
14	-0.3	5.4	8.5
15	0	5.3	8.5
16	5	4.6	8
17	5	4.5	8
18	5.1	4.5	8
19	5.1	4.5	8
20	5	4.5	8
21	10.1	3.9	7.5
22	10	3.9	7.5
23	10	3.8	7.5
24	9.9	3.7	7.5
25	10	3.8	7.5
26	15	3.2	7
27	14.7	3.2	7
28	15	3.2	7
29	15	3.2	7
30	15	3.2	7
31	19.8	2.9	6.8
32	20	2.9	6.8
33	19.5	2.8	6.8
34	19.5	2.8	6.8
35	19.5	2.8	6.8

## 2 Experimental Design, Materials and Methods

The experiments were carried out in the Laboratory of Refrigeration, Air Conditioning and Cryogenics (LRAC/PUC-Rio), from the Department of Mechanical Engineering at the Pontifical Catholic University of Rio de Janeiro.

**Fig. 1:**
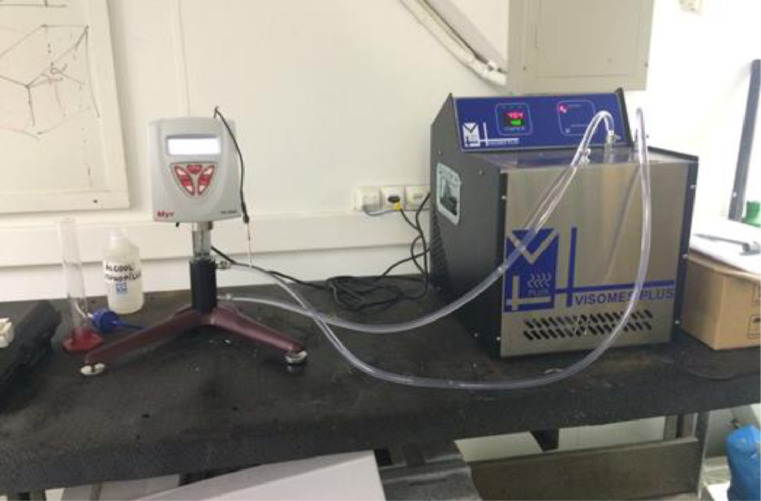
View of the experimental apparatus.

The experimental apparatus, depicted in Fig. 1, consisted of a thermostatic bath, a rotational viscometer (V2-L/ VR 3000 MYR), an adapter for small volume samples and flexible hoses that connected the thermostatic bath to the adapter. The V2-L viscometer was also equipped with a temperature sensor and a set of standard rods. The working fluid (responsible for the temperature stability of the nanofluid samples) circulated through flexible hoses from the thermostatic bath to the adapter. This technique provided good stability of the thermal conductivity enhancement.

The nanofluids that were used in the present work were produced at the Energy, Thermal Systems and Nanotechnology Laboratory (LEST-nano) at the Federal University of Uberlândia using the two-step technique, including the stabilization process carried out in a high-pressure homogenizer. The samples were delivered pre-dispersed by the supplier company Nanostructure d and Amorphous Materials (NanoAmor) and then diluted to obtain the desired concentrations of nanoparticles in the nanofluids.

1. Nanofluid I: Graphene nanoparticles at a concentration of 0.32% by mass were added to a mixture of ethylene glycol and distilled water at 30% by 70% by volume (base fluid II), respectively. In this solution, 50 layers of graphene sheets with an average length of 5-10 µm were used.
2. Nanofluid II: Graphene nanoparticles at a concentration of 0.2% by mass were added to a mixture of engine coolant (ASTM D-3306; ASTM D-4985; NBR 13705) and distilled water at 30% by 70% by volume (base fluid I), respectively. In this solution, 50 layers of graphene sheets with an average length of 5-10 µm were used.
3. Nanofluid III: Multi-walled carbon nanotubes at a concentration of 0.2% by mass were added to a mixture of engine coolant (ASTM D-3306; ASTM D-4985; NBR 13705) and distilled water at 30% by 70% by volume (base fluid I), respectively. The nanotubes had the following average dimensions: a diameter of 50 nm and a length within the range of 10-20 µm.

Soon after sample preparation, the actual concentration of nanofluids was verified by gravimetric analysis.

A viscometer V2-L/ VR 30 0 0 MYR, was employed to measure the viscosities of the base fluids plus the three nanofluids. It also included a temperature sensor and a set of standard rods. To ensure good temperature resolution (close to 0 . 1 °C) and stability (close to 0.5 °C), a thermostatic bath (Visomes Plus, VBT 2050) was used with power above 200 W.

I. Different temperature values ​​were defined under which measurements of the dynamic viscosity of the nanofluids and the shear stress exerted on them were performed. The temperature points adopted for the experimental tests were 9 (nine) in total: -15 ${}^{\circ }$0, -10 °C, -5 °C, 0 °C, 5${}^{\circ }$C, 10${}^{\circ }$, 15 °C, 20 ${}^{\circ }$C and 30 °C. The values ​​were chosen to consider the sensitivity of the more accentuated viscosity variation, namely at lower temperatures. Therefore, the interval between the points is 5 °C at the beginning of the temperature range and, from 20 °C, an additional increment of 10 °C was used to test at 30 °C. Furthermore, the lower bound temperature never exceeded -15 °C due to the operational limitation of the thermostatic bath and the solidification temperature of the fluid samples. Therefore, setting a certain temperature and waiting for the steady state to be reached in the system, the shear rate that was acting on the nanofluid samples was varied so that the measurements could be performed. Thus, after all measurements were performed, it was possible to construct the (viscosity vs temperature) and (shear stress vs temperature) relationships for a given shear rate.
II. By repeating the steps described in (i) for different shear rates, it was possible to obtain families of curves (each curve associated with a specific shear rate) of viscosity vs temperature and shear stress vs temperature.
III. Then, viscosity curves as a function of shear rate for constant temperature curves were constructed. In this way, another family of curves was obtained.
IV. All the steps above were repeated using different nanofluids and, thus, results were generated with new relationships between the same physical quantities for each nanofluid.

Two replicates were run for each experiment.

## Ethics Statement

Not applicable.

## CRediT Author Statement

**Guilherme Cunha Maia Nobre:** Writing – Original draft, Data validation, Formal analysis, **Luiz Umberto Rodrigues Sica:** Writing – review & editing, Writing – Original draft, Formal analysis; **Edwin Martin Cadenas Contreras:** Formal analysis; **Enio Pedone Bandarra Filho:** Funding acquisition, Project administration, Formal analysis; **Paul Ortega Sotomayor:** Methodology, Formal analysis; **José Alberto Reis Parise:** Funding acquisition, Project administration, Writing – review & editing.

## Declaration of Competing Interest

The authors declare that they have no known competing financial interests or personal relationships that could have appeared to influence the work reported in this paper.

## Data Availability

Dataset for viscosity of nanofluids and fluid base (Original data) (Mendeley Data).
